# Arsenic and Its Compounds

**Published:** 1862-08

**Authors:** Geo. Watt


### ARSENIC AND ITS COMPOUNDS.

BY GEO. WATT.

As a metal, arsenic, sometimes called *arsenicum*, is unimportant. Were it not that, by union with other elements, it forms important compounds, it would claim attention only as a curiosity in science.

The metal is readily obtained by decomposing arsenious acid, which is the well known white arsenic, or "rat's bane," of commerce. The method suggested by Turner is convenient for the dentist:—Mix a portion of white arsenic (arsenious acid) with two or three times its weight of powdered charcoal, put the mixture in a large Hessian crucible, invert a smaller one over it, and lute the two together. Heat the one containing the mixture to a red heat, while the other is kept comparatively cool. The metal sublimes, and is deposited on the inner surface of the upper crucible.

Arsenic is frequently found in combination with other metals, especially with iron, cobalt, and zinc. It is a brittle, whitish-gray, crystalline metal, having a strong metallic luster, and sublimes without liquefying, its vapor having the odor of garlic. It may be vaporized in close vessels without undergoing change; but when exposed to the atmosphere, it combines with oxygen.

With oxygen, this metal forms three compounds—a grayish *suboxyd*, *arsenious acid*, and *arsenic acid*. The composition of the first is not satisfactorily determined. It is supposed by some to be a mixture of arsenious acid with metallic arsenic. Arsenious acid is composed of one equivalent of arsenic united with three of oxygen. Its formula is, therefore, $AsO_3$, the degree of oxydation corresponding with that of sulphuric acid. Arsenic acid consists of one equivalent of arsenic combined with five of oxygen, its formula, $AsO_5$, corresponding with that of nitric acid. The equivalent of the metal being 75, and that of oxygen 8, it is easy to calculate the relative quantities of the two elements in any given quantity of either compound.

Arsenious Acid.—It is stated above that, when sublimed in contact with atmospheric air, arsenic united with oxygen. Arsenious acid is the compound usually formed. It seems to be the favorite combination of the two elements, and is, therefore, much more familiarly known than the others. Being so much more common than the other compounds, or than the metal uncombined, it is usually called, simply, "arsenic."

This acid may be melted, by heating it in a tube closed at both ends. By fusion, it becomes a colorless liquid. When left free, it volatilizes at about 400°, without melting, producing a colorless and inodorous vapor. Whenever the "garlic odor" is perceived, it is evident that the acid is, in part, at least, reduced, and that metallic arsenic is volatilized.

As commonly seen, arsenious acid is a fine white powder; but, in its solid state, it is found in three distinct modifications, one of them amorphous, and the others crystalline. When its vapor is condensed, at a temperature near its melting point, the acid is converted into a transparent, vitreous mass. When sublimed slowly, and the vapor is condensed at a lower temperature, the acid crystallizes in regular octohedrons. And crystals of the same form are obtained when the acid separates from an aqueous or ammoniacal solution. The acid is sometimes found in the form of thin, flexible, transparent plates, supposed to be derived from the right rhombic prism. The octohedral seems to be the favorite form with this acid, as the vitreous gradually passes into this modification, and the rhombic changes to it when volatilized.

The specific gravity of arsenious acid in its octohedral form is about 3.70, in its vitreous, 3.74. The vitreous is more soluble in water than the opaque, crystalline acid, though the observations of some experimenters lead to a different conclusion. According to Klaproth and others, 1,000 parts of water, at 60°, dissolve only two and a half parts of the powdered acid, while the same quantity of boiling water dissolves 77.75 parts, 30 of which it still retains when cooled to 60°.

The watery solution of arsenious acid is transparent, colorless, inodorous, nearly tasteless, and reddens litmus slightly.

This substance evinces its acid nature by forming definite salts with basic oxyds. One equivalent of the acid unites with two of lime, forming a subarsenite of lime, as represented in the formula, $2CaO, AsO_3$. With potash and soda it forms similar subarsenites, and also neutral arsenites, and hydrated binarsenites, the latter being represented by the formula, $KO, AsO_3$, and $KO, HO, 2AsO_3$.

Arsenious acid is more soluble in many acids than in water. From its solution in boiling hydrochloric acid, it crystallizes unchanged, the formation of the crystals being accompanied with the liberation of light.

The arsenites are all easily decomposed, and are all poisonous.

Arsenic Acid.—By the action of nitric acid, or *aqua regia*, arsenious acid is transformed into *arsenic acid*. This acid is readily obtained by dissolving arsenious acid in concentrated nitric acid and applying heat. The action is more prompt when a little hydrochloric acid is added. The mixture is then to be evaporated to dryness, to get rid of the excess of nitric and hydrochloric acids. Too high a temperature is to be guarded against, as at a red heat, or probably lower, the acid is decomposed, resulting in arsenious acid and oxygen. The action of nitric on arsenious acid is represented by the formula, $6AsO_3+4NO_5=6AsO_5+4NO_2$. The binoxyd of nitrogen, $NO_2$, escapes as a gas, and, as soon as it reaches the atmosphere, is changed to nitrous acid, $NO_4$, by taking oxygen from the air. The nitrous acid fumes are red or orange colored, and if an excess of nitric acid is used in the process, the cessation of these fumes indicates that all the arsenious acid in the mixture has undergone the desired change.

Arsenic acid, thus prepared, is a white, granular substance, has a sour, metallic taste, reddens litmus, is soluble in five or six times its weight of cold water, deliquesces slowly in the air, and from its solution thus formed, deposits hydrated crystals. With basic oxyds it forms definite salts, usually called *arseniates*, but correctly, *arsenates*.

ARSENIDE OF HYDROGEN.—When metallic arsenic is made the negative pole of a battery, in the galvanic decomposition of water, a solid compound of arsenic and hydrogen is formed, which possesses but little practical importance. But there is a gaseous compound of the same elements, which is called *arseniuretted hydrogen*, and which is worthy of attention. It is formed by the union of three equivalents of hydrogen with one of arsenic.

This gas may be obtained by various processes, but the most convenient is to dissolve zinc in dilute sulphuric acid in which arsenious acid is mixed. The reactions are represented as follows:

$$6Zn+3HO+AsO_3+6SO_3=6ZnO, SO_3+AsH_3.$$

It is well known that when zinc is dissolved in dilute sulphuric acid, hydrogen is liberated. In its nascent state, this element is able to take arsenic from any of its known combinations, and great reliance is placed on this fact in testing for arsenic, as will be noticed in due time.

Other compounds of arsenic are interesting, but are not of sufficient practical importance to claim special attention here. In a future number this subject will be continued.

